# Synovial chondromatosis of the temporomandibular joint: a case report with an associated systematic review update of the literature

**DOI:** 10.22514/jofph.2025.068

**Published:** 2025-12-12

**Authors:** Ovidiu Ionut Saracutu, Matteo Val, Luca Guarda-Nardini, Marco Ferrari, Matteo Pollis, Daniele Manfredini

**Affiliations:** ^1^Department of Medical Biotechnologies, School of Dentistry, University of Siena, 53100 Siena, Italy; ^2^Unit of Oral and Maxillofacial Surgery, Ca’Foncello Hospital, 31100 Treviso, Italy

**Keywords:** Synovial chondromatosis, Temporomandibular joint surgery, Arthroscopy, Case report, Systematic review, Treatment, Magnetic resonance imaging, TMJ pathology, Differential diagnosis

## Abstract

**Background**: This study aims to report a case of synovial chondromatosis (SC) of the left temporomandibular joint (TMJ) and provide an update of a previous review published in 2010 by summarizing the last 15 years of literature on the topic. **Methods**: On 07 July 2024, two of the authors independently performed a systematic search in the most relevant medical databases to identify all the English-language papers reporting cases of TMJ SC. After selecting the articles, the authors discussed their potential inclusion in the systematic review, and in case of disagreement, a third author was consulted. **Results**: From the assessment of all four databases, 466 citations were retrieved. After the removal of the duplicates, 136 articles remained for evaluation. In the end, a total of 108 articles were selected for inclusion, reporting a total of 1046 cases of SC of the TMJ. The most frequently reported signs and symptoms were pain, reduced interincisal mouth opening, swelling, crepitus, and clicking. **Conclusions**: Despite the different surgical treatment options that were adopted, the recurrence rate remains low. From the present review, no evidence arises for a specific risk factor for TMJ SC. **The PROSPERO Registration**: register number CRD42024568102.

## 1. Introduction

The World Health Organization (WHO) classified synovial chondromatosis (SC) as a rare, benign, locally aggressive bone tumor that can affect joints, bursae, and tendons [1]. SC originates from mesenchymal remnants of the synovial tissue and consists of the formation of multiple cartilaginous nodules that may be pedunculated and/or can detach from the synovial membrane. The first case report dates to 1558, when Ambroise Paré described it in a knee joint [2].

Despite many case reports being reported in the literature, the etiology of SC remains unknown. It is generically classified into primary SC and secondary SC, with primary cases being rarer and more aggressive than secondary ones, and not being associated with any known etiological factor. It generally affects males aged 20–50 years and has a prevalence of 1 in 100,000 [3]. Secondary SC cases occur in older individuals and have instead been associated with a history of trauma or pre-existing rheumatoid arthritis. However, no clear pathophysiological mechanism was hypothesized for this relationship. SC tends to affect large synovial joints, such as the knee, shoulder, hip, elbow, ankle, and wrist, while the temporomandibular joint (TMJ) is less commonly involved [4].

The main symptoms of TMJ SC are pain, reduced mouth interincisal opening, swelling, and crepitus sound. Such symptoms are actually nonspecific since they are common clinical findings in patients who seek for advice due to the potential presence of temporomandibular disorders (TMD) [5, 6, 7]. Thus, diagnosis is often challenging and requires the aid of second-level instrumental investigations via imaging, such as magnetic resonance imaging (MRI) and computerized tomography (CT) [8]. MRI of SC patients unveils the presence of one or multiple nodules in the joint space, usually with a considerable amount of effusion [9], whilst CT allows a better estimate of the potential changes to the TMJ bony structures [10]. However, the histological examination must further confirm SC diagnosis to rule out the presence of other pathologies [11].

In 1977, Milgram described the histopathologic findings and proposed a classification based on three stages: (i) Active SC lesion with the absence of loose bodies, (ii) transitional lesions with both the presence of active SC lesion and free bodies, (iii) multiple free bodies with the absence of metaplasia [12]. Some years later, the same three types of histopathological findings of SC affecting the TMJ were described by Blankestijn *et al*. [13], confirming the temporal sequence of the developmental stages.

The treatment of SC is based on the surgical or arthroscopic removal of the multiple cartilaginous nodules and loose bodies from the temporomandibular joint space with or without synovectomy. Arthroscopy showed a lower success rate than open surgery when used alone [5].

The first case report of TMJ SC was published by Axhausen in 1993 [14]. Since then, many case reports and some associated systematic reviews have been reported in the literature, but most of them did not follow the Preferred Reporting Items for Systematic Reviews and Meta-Analyses (PRISMA) guidelines for systematic reviews. Two previous systematic reviews tried to summarize the main findings: Von Lindern *et al*. [15] reported all the cases of SC present in the literature up to 1997, and a decade later Guarda-Nardini *et al*. [4] updated the review with a total of 80 cases of SC. In recent years, the various systematic reviews that have been published on the topic of TMJ SC had specific inclusion and exclusion criteria and did not provide a comprehensive summary of the topic that was comparable to the two early reviews.

Based on these premises, the present paper aims to describe a case of SC of the TMJ and to provide an update on the previous review by Guarda-Nardini *et al*. [4], summarizing the main findings of all the SC cases published in the last 15 years. The case report was written according to the CARE (Case Reports) guidelines, while the PRISMA guidelines were followed for the systematic review, as documented in the **Supplementary material 1** [16].

## 2. Case report

On 30 May 2023, a 51-year-old woman was referred to the University of Siena Orofacial Pain Unit because of the presence of TMD symptoms. The patient reported presence of left TMJ pain in the last two years, with occasional swelling and reduced mouth opening. The patient denied a previous history of trauma or diagnosis of any rheumatic conditions. Clinical examination revealed the presence of pain upon palpation of the left TMJ and the main ipsilateral tendon insertions. The patient also presented reduced mouth interincisal opening (MIO = 30 mm). The contralateral joint was negative upon palpation. Extraoral inspection revealed the presence of a cutaneous swelling around the articular capsule (Fig. 1). The patient did not report any dysfunction to the facial nerve or the vestibular or auditory system. No joint sound was present upon condylar translation. The clinical examination suggested the need for further diagnostic investigations using MRI and CT.

**Fig. 1.** Pre-operative extraoral picture. Patient presents cutaneous swelling (red circle) at the level of the left TMJ.

MRI showed the presence of four intra-articular free bodies, apparently wrapped into a cystic neoformation. The free bodies displayed characteristics of moderately voluminous nodules (Fig. 2a,b).

**Fig. 2.** Magnetic resonance imaging. (a) Sagittal view of the left TMJ. It is possible to appreciate the four free loose bodies (red arrow). (b) Coronal view of the MRI, showing the presence of calcified free bodies within the left TMJ space (red arrow).

CT confirmed the presence of at least two of the four free bodies within the left TMJ space (Fig. 3). CT also showed the overall integrity of the condylar cortical structure, with just some signs of erosion of the condylar neck, probably due to the compression caused by the loose bodies. The diagnostic hypothesis of SC of fibrous type of the left TMJ was formulated. The possible differential diagnosis to consider was pseudogout of the TMJ or neoformation of auto-immunity origin (*e.g.*, heterotopic bone formation due to mixed connective tissue disease). The patient received information about the nature of the disease, the prognosis, and the treatment options. Eventually, the patient agreed to perform the surgery. The patient was then referred to the Department of Maxillofacial Surgery of Ca’Foncello Hospital, Treviso, Italy, for the surgical intervention.

**Fig. 3.** Axial view of the CBCT. Presence of calcified loose body (red arrow).

An open surgery approach was chosen due to the ease of removing eventual wide loose bodies within the joint. Under general anesthesia, the left TMJ was exposed by a preauricular incision (Fig. 4). After exposure, the joint capsule was exposed, six large calcified loose bodies were found within the TMJ and were removed (Fig. 5). Three smaller calcified free bodies were found in the superior joint space and were removed as well. Additionally, four fibrous micronodular bodies were found at the level of the condyle. Complete synovectomy was performed via the open surgery approach to remove any residuals from the TMJ with the potential for further metaplastic activity. Attention was placed to preserve the integrity of the articular disc. Intraoperatively, the TMJ disc did not appear involved by the SC and was in the clinically normal position with no perforation.

**Fig. 4.** Preauricular access to the left TMJ.

**Fig. 5.** Removal of a large calcified loose body from the TMJ space.

The histopathological examination confirmed the diagnosis of Stage 3 SC of the left temporomandibular joint according to Milgram classification [12] and excluded the presence of other pathologies (Fig. 6). The histological findings also confirmed the presence of calcifications.

**Fig. 6.** Histopathological finding showing chondrometaplasia of the synovial membrane. Fragment of mature cartilaginous tissue with isolated clusters of endochondral ossification.

The postoperative course was uneventful, and a regimen of painkillers and antibiotics was prescribed. After the surgery, no motor deficit on the left side of the face was present (Fig. 7). The patient performed one month of physiotherapy, and a series of follow-up assessments were scheduled at one week, one month, three months, six months, and one year. At the one-year follow-up, the patient came to the attention of the same operators, who had been in charge of the surgical intervention, to undergo the clinical examination. No pain was reported upon palpation, function, and orthopaedic test. The MIO was 39 mm. Given the lack of any signs or symptoms of recurrence, it was decided to keep the patient under periodic control at one-year intervals. Considering the resolution of symptoms, it was not considered necessary to perform an MRI at the one-year follow-up.

**Fig. 7.** Extraoral picture taken one-week post-surgery.

## 3. Materials and methods for the systematic review

### 3.1 Inclusion and exclusion criteria

This systematic review adhered to the guidelines outlined in the Preferred Reporting Items for Systematic Reviews and Meta-Analyses (PRISMA) (**Supplementary material 1**). The eligibility criteria of the present study were based on the PEO acronym (Population, Exposure, Outcome) for qualitative research question, in which P: patients with a diagnosis of chondromatosis of the TMJ, either bilateral or unilateral; E: Imaging/histological findings and/or surgical treatment of chondromatosis; O: prevalence of the condition and/or improvement in symptomatology, functionality, and quality of life, responsiveness to treatment.

Thus, to achieve comprehensiveness, given the rarity of the condition, all studies reporting at least one case of SC were included in the review.

The exclusion criteria were: (1) Studies that did not include at least one case of chondromatosis; (2) Studies where a definitive diagnosis of chondromatosis was not reached; (3) Studies on animals; (4) Reviews, letters, book chapters, conference papers, posters, and guidelines; (5) Studies with duplicated data from another included study; (6) Full-text not available even after contacting the first authors (4 weekly attempts in one month); (7) Studies that were included in the previous systematic review by Guarda-Nardini *et al*. [4]; and (8) Studies that were written in non-English language. The systematic review was registered in PROSPERO (International database of prospectively registered systematic reviews in health and social care) with the register number CRD42024568102. The authors certify that they have obtained all appropriate patient consent forms in accordance with the Helsinki Declaration and understood that the patient was free to withdraw from the study at any time. In the form, the patient has given her written consent for her images and other clinical information to be reported in the journal. The protocol for studying temporomandibular disorders and their surgical treatment was approved in 2024 at the Maxillofacial Surgery Unit of Ca’Foncello Hospital in Treviso (Italy) (Ethical Committee approval numbered 581/CE Marca).

### 3.2 Information sources

On 07 July 2024, two of the authors of the paper (OIS, MV) independently performed a systematic search in the four most relevant medical databases: National Library of Medicine’s PubMed Database, Scopus, Web of Science, and Cochrane Library, to identify all the English-language papers reporting cases of TMJ SC. An expert librarian of the University of Siena assisted the three authors during the process. The reference lists of the included articles were checked to search for additional articles. Grey literature was not searched.

### 3.3 Search strategy

No age limit was set. No filter was used in any of the consulted databases. The papers in other languages than English were excluded by the authors when assessing the whole list of articles. The search was limited to articles published later than 01 January 2009, and the papers already included in the previous systematic review by Guarda-Nardini *et al*. [4] were not considered. The following search strategy was adopted; (i) a first search in PubMed using the Medical Subjects Headings (MeHS); (ii) a second search in PubMed using free words; (iii) a search assessing the related articles of PubMed; (iv) a search using free words in the Scopus, Web of Science, and Cochrane databases; (v) a search within the reference lists of the included articles;

In PubMed, the following MeSH terms were used to identify the list of potential articles to be included:

● TMJ: An articulation between the condyle of the mandible and the articular tubercle of the temporal bone (MeSH Unique ID: D013704);

● SC: Rare, benign, chronic, progressive metaplasia in which cartilage is formed in the synovial membranes of joints, tendon sheaths, or bursae. Some of the metaplastic foci can become detached, producing loose bodies. When the loose bodies undergo secondary calcification, the condition is called synovial osteochondromatosis (MeSH Unique ID D015838). The following search strategy was used in all the databases: Temporomandibular Joint AND Synovial Chondromatosis.

### 3.4 Selection process

Three authors (OIS, MP, MV) initially planned the complete search strategy before proceeding with the assessment on their own. Two of them first screened the articles by reading the title and the abstract. After selection of the articles, the authors discussed the papers to be include in the review. In case of disagreement, a third reviewer (MP) was consulted. In the end, a final consensus was reached. After the retrieval of the articles from the databases, Mendeley (Mendeley Ltd, London UK) was used to eliminate duplicates and obtain a unique list of potential articles.

### 3.5 Data collection process and data items

The authors who performed the systematic research (OIS and MV), after agreeing on the variables to extract from the included articles, created a common table template using Microsoft Office Excel 2021 (Microsoft, Los Angeles, CA, USA) that they used independently to extract the data from the included articles. At the end of this step, the authors discussed the discrepancies between their tables and solved them, if needed. For each included article, the following data were extracted: sample size, age, gender, affected joint (right, left, or both), clinical signs and symptoms, history of trauma, history of rheumatoid arthritis or other autoimmune diseases, duration of signs and symptoms, diagnostic imaging technique, type of surgical technique, number of loose bodies extracted from the TMJ space, follow-up span, adjuvant therapy, and recurrence. In case one of any missing information, the corresponding box of the table was left empty. Finally, a table with all the articles included was created. No automation tools were used in the whole process of article retrieval and data extraction.

### 3.6 Study risk of bias assessment

The methodological quality of the included papers was assessed using the Joanna Briggs Institute (JBI) Critical Appraisal Checklist for case reports and case series (Fig. 8) [17]. This tool is composed of 8 items for case reports and 10 items for case series, which evaluate the quality of the papers. The ratings were given by the authors of the review to assess the risk of bias due to possible flaws present in the studies.

**Fig. 8.** Joanna Briggs institute critical appraisal checklist.

For this review, an overall assessment was performed for each included study. The authors of the review discussed collegially to decide the key items that ensure a high methodological quality of the paper. The articles that presented all these elements were categorized as having low risk of bias, compared to those with negative or uncertain ratings.

Given the scope of this systematic review and the rarity of the condition investigated, items #1, #2, #3, #4 of the Joanna Briggs Institute Critical Appraisal Checklist for case reports and case series were considered of critical importance. The studies that missed at least one of the four items identified as fundamental for the purpose of this review were categorized as having a high risk of bias. The articles that had all the items were instead categorized as having a low risk of bias. All the other cases were marked as unclear risk of bias.

### 3.7 Effect measures and synthesis methods

Given that in the present review only case series and case reports were included, it was not possible to perform a meta-analysis nor any type of statistical analysis.

## 4. Results

### 4.1 Study selection

The first search strategy with the MeSH terms yielded 63 citations. The second search step of the search strategy was performed using the combination of free words “Temporomandibular joint” and “Synovial chondromatosis” and led to 140 citations. The same strategy repeated in the Scopus database and Web of Science led to 158 and 105 citations, respectively. Conversely, the Cochrane Library did not yield any citations. No additional paper potentially relevant to the aim of the systematic review was retrieved from the PubMed related articles. Moreover, the search within the reference lists of the included articles did not lead to the identification of additional papers.

From all four databases, a total of 466 articles were retrieved. After the removal of the duplicates, 136 articles were left. Of them, six articles were excluded from abstract reading. Of the remaining 130 papers, 108 met the inclusion criteria, and their data were extracted for the purposes of this review. Fig. 9 shows the PRISMA flowchart for the systematic review process. Table 1 summarizes the main findings of the systematic review, while **Supplementary material 2** provides a detailed overview of the included articles [18, 19, 20, 21, 22, 23, 24, 25, 26, 27, 28, 29, 30, 31, 32, 33, 34, 35, 36, 37, 38, 39, 40, 41, 42, 43, 44, 45, 46, 47, 48, 49, 50, 51, 52, 53, 54, 55, 56, 57, 58, 59, 60, 61, 62, 63, 64, 65, 66, 67, 68, 69, 70, 71, 72, 73, 74, 75, 76, 77, 78, 79, 80, 81, 82, 83, 84, 85, 86, 87, 88, 89, 90, 91, 92, 93, 94, 95, 96, 97, 98, 99, 100, 101, 102, 103, 104, 105, 106, 107, 108, 109, 110, 111, 112, 113, 114, 115, 116, 117, 118, 119, 120, 121, 122, 123, 124, 125]. In particular, **Supplementary material 2** encompasses data from all the articles that have been included in the systematic review, specifying for each of them the number of cases included, the demographic features, the signs and symptoms, the diagnostic imaging techniques, the surgical techniques, the number of loose bodies that were extracted, the duration of follow-up, the adjuvant therapy, and the recurrence rate.

**Fig. 9.** PRISMA flow-chart of the systematic review.

**Table 1.** Main findings of the systematic review.

Main Findings		N (%)
Study Characteristics		
	Articles Included	108
	Number of cases reported	1046
	Mean age (yr)	61.8, range 21–82
Patients with reported signs and symptoms		503
	Pain	400 (79.5%)
	Reduced interincisal opening	260 (51.7%)
	Reported swelling	173 (34.4%)
	Crepitus	50 (9.9%)
	TMJ click	25 (5.0%)
Risk of bias of the included studies		108
	High risk of bias	65 (60.2%)
	Unclear	11 (10.2%)
	Low risk of bias	32 (29.6%)
Patients with diagnostic imaging documentation available		836
	Orthopantomography	117 (14%)
	Magnetic Resonance Imaging	751 (89.8%)
	Computerized Tomography	260 (31.1%)
	Cone-Beam Computerized Tomography	155 (18.54%)
Patients with the report of the surgical procedure		629
	Open surgery	456 (72.5%)
	Arthroscopy	172 (27.3%)
	Intraoral approach	1 (0.2%)

*TMJ: temporomandibular joint.*

### 4.2 Study characteristics

The included articles reported a total of 1046 cases of SC of the TMJ. Within the included papers, 39 of them reported more than one case, contributing to the high number of patients reviewed. Demographic characteristics were available only for 703 patients. The patients’ mean age was approximately 61.8, ranging from 21 to 82 years. All but five studies specified the gender of the patients, for a total of 575 cases from 103 papers. The number of females affected by SC (401) was more than double compared to males (173). 95 studies specified the side of the SC lesions (right side, n = 256, left side, n = 248, bilateral sides, n = 5).

### 4.3 Risk of bias in studies

Of the included studies, 65 (60.2%) had a high risk of bias. The studies were either missing an accurate description of the demographic characteristics of the patients, a timeline of the whole clinical process, a detailed description of the case, or an accurate description of the diagnostic tests. Moreover, 11 studies (10.2%), despite having all the items that were considered to be critical, were missing information regarding all the other aspects of the JBI Critical Appraisal Checklist. The other 32 studies (29.6%) were considered to be at a low risk of bias. Nevertheless, it is important to consider that in general, case-series and case-control investigations are designs with a low level or quality of evidence compared to other types of studies.

### 4.4 Results of individuals studies and of synthesis

The reported signs and symptoms were specified by 94 studies, for a total of 503 cases of TMJ SC. They were, in order of frequency: pain, reduced mouth interincisal opening (MIO), swelling, tenderness, crepitus, click, and, in some rare instances, headache. Indeed, of the 503 patients with documented signs and symptoms, 400 (80%) reported pain, 260 (52%) reported reduced interincisal opening, 173 (34%) reported swelling, 50 (10%) reported crepitus, 24 (5%) reported presence of click. Only 15 patients reported a history of trauma, and only 4 were affected by rheumatoid arthritis. The duration of signs and symptoms was heterogeneous, ranging from 15 days to 25 years.

#### 4.4.1 Diagnostic imaging techniques

Of the total number of patients in the systematic review, 836 (80%) underwent a radiographical or magnetic resonance examination. Of them, 751 (72%) underwent MRI, 260 (25%) CT, and 155 (15%) CBCT (Cone Beam Computer Tomography), while 117 (11%) performed an OPT (orthopantomography).

#### 4.4.2 Surgical techniques and number of loose bodies

Most of the studies (82%) described the surgical intervention that was performed, for a total of 629 patients. 456 (72%) of them underwent open surgery intervention, while 172 (27%) received arthroscopic intervention, and only one case was treated with an intraoral approach. The loose bodies removed from the TMJ vary in size and number. In 17 patients, one single mass was found. However, in other cases, the number of loose bodies was higher, with the most peculiar cases reporting 180 bodies. The biggest mass was 50 × 55 × 40 mm in size. In most papers, the authors did not specify the number of loose bodies that were removed, but they only reported the removal of multiple bodies. In general, no clear information was provided about the exact location of the loose body at the level of the TMJ. Two studies reported the involvement of the cranial fossa, requiring a multidisciplinary approach to surgical management [66, 75].

#### 4.4.3 Follow-up, adjuvant therapies and recurrence rate

Of the 108 studies included in the review, 62 provided data concerning the follow-up, which ranged from two weeks to almost 10 years. Of the 319 patients who had a follow-up, only 20 had a recurrence of SC (6%) and needed a second intervention [23, 86, 115, 122, 124]. Nevertheless, the data must be interpreted with caution, as many authors do not have a follow-up of the presented case. It was not possible to assess if the follow-up duration could have influenced the recurrence rate since some of the authors that reported recurrence did not specify its timing. Interestingly, the cases of recurrence were reported in the papers where TMJ arthroscopy was adopted for the removal of loose bodies.

After the surgery, some authors proposed the use of adjuvant therapies to lessen the post-operative discomfort. Eight authors proposed physiotherapy, and two opted for physiotherapy in combination with a Michigan splint. Non-steroidal anti-inflammatory drugs were also prescribed in three cases. One author suggested using the splint, and one performed infiltration of hyaluronic acid 30 and 60 days after the surgical intervention. In all the cases, the post-operative course was uneventful.

Since it was not possible to perform a meta-analysis, no synthesis of results was reported.

## 5. Discussion

Synovial chondromatosis of the temporomandibular joint is a rare condition. Most of the articles presented in the literature are case reports of single or multiple cases. Two relevant systematic reviews were published on the topic [4, 15] with the same attempt to summarize all the literature of the previous decades. The present study aimed to provide an update to the previous systematic reviews, reporting all the cases of TMJ SC that were published in the last 15 years. Some of the articles included in the current review were also attempts to perform a systematic literature review accompanying the description of a case report [22, 25, 27, 50, 51, 62, 69, 79, 85, 96, 97, 101, 104, 113, 114, 117]. However, none of them followed the PRISMA checklist and met the best possible standards of literature review. The systematic assessment of medical databases led to a total of 108 studies, with 1046 cases of TMJ SC. Most of the included articles were case reports or case series describing the main clinical features of the patients. Unlike other reviews, our manuscript includes all the papers that mentioned a case of synovial chondromatosis, not just the articles providing full documentation of the patients. Such a strategy was adopted to provide a clearer representation of the prevalence of TMJ SC and provide a better description of the epidemiological picture.

In the literature, there are more case reports of TMJ SC in females compared to males, and the average age is 61 years. This data about a possible female predisposition to the disease is in contrast with the literature on the SC of the whole body, which affects males 1.8 to 3 times more than females [126]. Indeed, there is little knowledge of the etiology of this disease, and the only risk factors that have been associated with SC are a history of trauma and rheumatoid arthritis [126]. Despite this, in the present paper, only 15 patients reported a previous trauma, and only four were also diagnosed with rheumatoid arthritis. Until now, no study has demonstrated an association between the onset of SC and possible risk factors. Such shortcomings might also be due to the nature of the case reports and case series, which are generally characterized by a high risk of bias as far as any speculation on etiology is concerned, and do not have the adequate study design to investigate the importance of specific risk factors [127]. All the data reported in the conclusion are largely influenced by the high risk of bias reported in most of the papers due to the lack of information on the topic, and they must be interpreted as a picture of the available evidence rather than as the absolute epidemiological overview of the TMJ SC. Another limitation that prevents us from getting deeper into the association between TMJ SC and risk factors is that only half of the patients included in this review had adequate information concerning their history.

The most frequently reported signs and symptoms were pain (80%), reduced mouth opening (52%), and swelling (34%), which are similar to what is reported in SC of larger joints [126]. Such symptoms are unspecific, and they can be the cause of a late diagnosis, as they are not different from the common symptoms of TMD patients. However, some other additional and more specific findings, such as the presence of psychological impairment [128, 129, 130, 131, 132, 133, 134, 135] and otologic symptoms such as tinnitus [136], can help clinicians to perform a differential diagnosis prior to requesting a second-level diagnostic imaging technique [137, 138]. Another possible hint that TMD might not be the cause of the impairment is the absence of response to any conservative treatment strategy [139, 141, 142, 143]. Indeed, some of the patients reported in the studies of this systematic review had received a misdiagnosis of TMD. As a result, they did not experience any benefit from the routine treatments, such as behavioral strategies [143], use of oral appliances [144], and non-steroidal anti-inflammatory drugs [145]. In such cases, advanced imaging techniques, such as MRI and CT, can help clinicians to detect the presence of loose bodies within the TMJ, suggesting a possible diagnosis of SC [121]. MRI can be particularly useful in determining the presence of nodules before ossification and in planning the surgical intervention [27]. Both CT and MRI should actually be mandatory diagnostic investigations prior to the surgical treatment of TMJ SC, since they provide the surgeon with the necessary information to decide the type of surgical intervention and previsualize the location of all the loose bodies [121].

Surgery is the treatment of choice for TMJ SC. The open surgery approach was the most frequently performed intervention associated with synovectomy and condyloplasty, based on the extension of SC. Some authors also proposed arthroscopic procedures with a satisfactory success rate [22, 23, 26, 37, 38, 43, 49, 56, 58, 64, 68, 70, 73, 85, 86, 87, 109, 113, 114, 115, 119, 120, 122, 124, 125]. The characteristics of the studies included in the review do not allow for an understanding of which type of intervention provides the best clinical outcomes. However, it is important to note that in studies adopting the open surgery approach, no recurrence was reported; relapses were reported only in a few patients who underwent arthroscopy [23, 86, 115, 122, 124]. The authors of the previous systematic review reported a lower success rate of arthroscopy, around 55%, with a need for a second surgical intervention [4]. One study [125] proposed using arthroscopy as a first-line strategy and open surgery as a second option in case of recurrence. However, no evidence supports such a strategy, which potentially exposes the patients to two interventions (*i.e.*, overtreatment). Before the intervention, patients should be informed about all the possible treatment options and of the risk of a second intervention in the case arthroscopy fails to remove all the loose bodies from the TMJ space. Interestingly, some patients refused the surgical intervention and opted for a wait-to-see policy, preferring to keep the disease under monitoring [34, 81]. Patients should be informed that such strategies, despite not having negative consequences in the short term, will only delay the intervention, as SC cannot be treated without surgery. Moreover, a long waiting time after the diagnosis can inevitably lead to a growth of the lesions, as demonstrated in a 7-year observational follow-up study [34]. The extension of SC can even spread to the cranial base [23, 96] and make the surgical intervention more complicated, often requiring the aid of a neurosurgeon. Despite this, patients should be aware that SC rarely progresses to malignancy. In the literature, there is only one case of chondrosarcoma associated with SC [48], and no data are available on the prevalence and incidence of the TMJ SC evolution toward malignancy. However, it is unclear if chondrosarcoma is raised from the SC lesion or as a comorbidity. Several similarities exist between this paper and the previous systematic reviews [4, 15]. However, the present study contains more than double the number of papers and a thirteen times higher number of patients. Despite this, the review contains the same shortcomings as the previous ones, mainly related to the often-insufficient documentation of clinical cases. Many studies lack an adequate evaluation of pain intensity based on numerical scales, a description of the imaging outcomes, and the disease staging based on the histological exams based on the Milgram classification [12]. These findings not only suggest a need for better-structured case report studies with adequate documentation, possibly following the CARE guidelines [16] but also invite the editors and the reviewers of scientific journals, before acceptance of the papers, to make sure that case reports contain the necessary documentation.

The findings of the present paper confirm the results of the previous systematic review by Guarda-Nardini *et al*. [4], despite the much higher number of cases reported in the literature in the last 15 years. In particular, the previous review found a 2.5:1 female/male ratio while the present paper found a 2.3:1 ratio. The mean age of SC was, however, higher in the present work (*i.e.*, 61 *vs*. 46 years). Similarly to the previous review, the number of right side TMJ SC cases was slightly higher than the left side ones. Interestingly, in the 2009 systematic review, only one case of recurrence was reported, whilst in the present work they were 20. Recurrence was reported in papers where the surgical procedure was performed via arthroscopy, possibly due to the difficulty to remove all loose bodies compared to the open surgery approach. Despite the recurrence rate remaining generally low, patients with recurrence of SC always require a second intervention; thus, this aspect should be discussed with the patients prior to the intervention and the possible proposal of an arthroscopic procedure. In this regard, future studies with a prospective design are needed to better predict the recurrence rate of both techniques.

The findings of the present systematic review are limited by the design of the included studies, which were only case reports and case series. Another limitation is related to the high heterogeneity of the methodology among the papers, which does not allow for a meta-analysis of the data. Moreover, more than half of the studies were considered to have a high risk of bias due to limited description of the case reports.

## 6. Conclusions

In the present study, a case report of SC of the TMJ has been reported and a systematic literature review of the past 15 years has been performed. In total, 1046 cases of TMJ SC have been documented, with a female/male ratio of 2.3:1 and a mean age of 61 years. Only five cases had bilateral involvement of the TMJ. Due to the non-specific symptoms, diagnosis of SC is often delayed and is achieved thanks to second-level diagnostic imaging techniques. Open surgery remains the treatment of choice, and additional procedures such as synovectomy, diskectomy, and condylectomy might be necessary. The recurrence rate is low, with only 20 cases reported in the literature, all of them reported after arthroscopic removal of loose bodies. From the included studies, no evidence arises on the etiology or the risk factors of TMJ SC.

## Supplementary Material

Supplementary material associated with this article can be found, in the online version, at https://files.jofph.com/ files/article/1999364025790414848/attachment/ Supplementary%20material.zip.




