# Supplementary material for: Synovial chondromatosis of the temporomandibular joint: a case report with an associated systematic review update of the literature
Source: J Oral Facial Pain Headache. 2025 Dec 12;39(4):70–84. doi: 10.22514/jofph.2025.068 (PMC12727185; doi:10.22514/jofph.2025.068)
Supplement: Supplementary file 2 [file Supplementary-material-2.docx]

Supplementary material 2

Supplementary Table 1. Summary of data from the articles included in the systematic review.

| Study’s first author and year | Sample Size | Mean Age (yr) | Sex (M/F) | R/L joint | Clinical signs and symptoms | History of trauma | History of rheumatoid arthritis | Duration of signs and symptoms | Diagnostic imaging techniques | Surgical technique | Number and size in mm of loose bodies | Follow-up duration (mon) | Adjuvant therapy | Recurrence | Risk of bias |
| --- | --- | --- | --- | --- | --- | --- | --- | --- | --- | --- | --- | --- | --- | --- | --- |
| 1. Murakami K, 2009 [18] | 1 | 53 | 1 M | 1 L | Pain, reduced MIO (36 mm), swelling, dullness | No | No | 6 months | OPT, CT, MRI | Open Surgery with diskectomy and synovectomy. Arthroplasty of the mandibular condyle | 46 (1 to 4 mm diameter) | 60 months | N.S. | No | Low |
| 2. Balasundaram A, 2009 [19] | 1 | 82 | 1 M | 1 L | Reduced MIO (35 mm), swelling, tenderness | Yes | No | 5 years | OPT, CT | - | - | - | - | - | High |
| 3. Sato J, 2010 [20] | 1 | 74 | 1 F | 1 L | Pain, reduced MIO (35 mm), swelling | No | No | 10 years | CT, MRI | Open Surgery | 180 bodies, 1–5 mm | 30 months | N.S. | No | Low |
| 4. Goizueta-Adame CC, 2010 [21] | 2 | 15, 74 | 2 F | 2 L | Pain (2/2), reduced MIO (29 mm) (1/2), (14 mm) (1/2) | No (2/2) | No (2/2) | 4 years (1/2), N.S. (1/2) | CT, MRI (1/2), OPT (1/2) | Open Surgery and costochondral graft and alloplastic prosthesis (2/2) | Single mass (2/2) | 12 months (1/2) 18 months (1/2) | Physiotherapy (1/2) | No | High |
| 5. Chen MJ, 2010 [22] | 1 | 50 | 1 M | L | Pain (70 on VAS), reduced MIO (max 28 mm), pre-auricular swelling, crepitus | No | No | 10 months | OPT, CT, MRI | Arthroscopy | Multiple (unspecified) | 18 months | NSAID and soft diet for 3 weeks | No | Low |
| 6. Meng J, 2010 [23] | 20 | median 44, range 32–67 | 12 F, 8 M | R (12/20) L (7/20) Bilateral (1/20) | Pain (17/20), reduced MIO (14/20), preauricular swelling (13/20), crepitus (5/20), headache (3/20) | Yes (1/20) | No | 2 months to 20 years (median 3 years) | Conventional radiography (20/20), CT (15/20), MRI (13/20) | Open Surgery (19/20), Arthroscopy (1/20) | Multiple (unspecified) | N.S. | N.S. | Yes (4/20), No (16/20) | High |
| 7. Boffano P, 2010 [24] | 1 | 55 | 1 F | 1 R | Pain, swelling, crepitus | No | N.S. | N.S. | CT | Open Surgery, arthrotomy and synovectomy | Multiple (unspecified) | 6 months | Physiotherapy | No | High |
| 8. Pimenta e Souza D, 2010 [25] | 1 | 28 | 1 M | 1 R | Swelling, crepitus | No | N.S. | Incidental diagnosis | OPT, CT | Open Surgery | Multiple (unspecified) | 24 months | N.S. | No | High |
| 9. Cai XY, 2010 [26] | 1 | 48 | 1 F | 1 L | Pain, reduced MIO (37 mm), preauricular tenderness, click | No | No | N.S. | MRI | Arthroscopy | 1 (16 × 9 mm) | 60 months | N.S. | No | High |
| 10. Testaverde L, 2011 [27] | 8 | Mean 39 years, range 18–67 | 3 M, 5 F | - | - | - | - | - | CT and MRI | - | - | - | - | - | High |
| 11. Gonzalez-Perez LM, 2011 [28] | 1 | 50 | 1 M | L | Pain, reduced MIO, swelling, crepitus | Yes | No | 2 years | OPT, CT, MRI | Open Surgery with condylar shaving | 35 | 24 months | Physiotherapy and Michigan splint | No | Low |
| 12. Guijarro-Martinez R, 2011 [29] | 1 | 38 | 1 F | Bilateral | Pain, preauricular swelling, headache, click, effusion | No | No | 1 year | CT, MRI | Open surgery with synovectomy and meniscopexy | Multiple (unspecified) | 36 months | NSAID, analgesics and soft diet | No | Low |
| 13. Varol A, 2011 [30] | 1 | 46 | 1 M | L | Pain, joint noise, tenderness | Yes | No | N.S. | OPT, CT, MRI | Open Surgery with partial synovectomy and disk repositioning | 15 | 24 months | No | No | Unclear |
| 14. Campbell DI, 2011 [31] | 1 | 59 | 1 M | 1 L | Pain, swelling, reduced MIO (20 mm) | No | No | 3 days | MRI | Open Surgery with temporalis muscle flap rotated into the joint space | Multiple (unspecified) | 3 months | N.S. | No | Low |
| 15. Shah SB, 2011 [32] | 1 | 25 | 1 F | 1 L | Pain, reduced MIO (32 mm) | No | N.S. | 2 years | CT | Open Surgery with diskectomy and synovectomy. Arthroplasty of the mandibular condyle | 43 | 48 months | N.S. | No | Low |
| 16. Fuller E, 2011 [33] | 1 | 45 | 1 M | 1 L | Tenderness | N.S. | N.S. | 10 years | CT, MRI | - | - | - | - | - | High |
| 17. Mori Y, 2011 [34] | 1 | 52 | 1 F | 1 L | Crepitus | No | No | N.S. | CT, MRI | - | - | - | - | - | High |
| 18. Lim SW, 2011 [35] | 1 | 49 | 1 M | 1 R | Pain, reduced MIO (27 mm), swelling, tenderness, click | No | No | 5 days | OPT, CT, MRI | Open Surgery | Multiple (unspecified) | 10 months | N.S. | No | Low |
| 19. Phelan E, 2011 [36] | 1 | 43 | 1 F | 1 L | Pain, trismus, swelling | N.S. | N.S. | 10 years | CT, MRI | Open Surgery | Multiple (unspecified), 1–5 mm diameter | 0.5 month | N.S. | No | Unclear |
| 20. Cai XY, 2012 [37] | 33 | Average 43, range 21–62 | 26 F, 7 M | 18 R, 15 L | Pain, trismus, reduced MIO (16/33), tenderness (20/33), joint sound (18/33), preauricular swelling (3/33), effusion (13/33) | N.S. | N.S. | Mean 13 months, range (2–72 months) | MRI (33/33), CT (4/33) | Arthroscopy (33/33) | up to hundreds | 3 to 108 months (mean 38) | N.S. | No | Unclear |
| 21. Matsumoto K, 2012 [38] | 1 | 34 | 1 F | 1 R | Pain | No | N.S. | 1 month | CT, MRI | Arthroscopy and partial synovectomy | Multiple (unspecified) | 12 months | N.S. | No | Unclear |
| 22. Jiang B, 2012 [39] | 1 | 39 | 1 M | 1 R | Pain, reduced MIO (35 mm), swelling, crepitus | No | N.S. | 12 months | CT, MRI | Open Surgery | Hundreds (1–10 mm in size) | 44 months | N.S. | No | Unclear |
| 23. Chen MJ, 2012 [40] | 5 | 50, 29, 30, 41, 35 | 1 F, 4 M | L (3/5), R (2/5) | Pain (4/5), crepitus (2/5) | No | No | 10 Months (1/5), 1 year (2/5) 2 Years (2/5) | CT, MRI | Open Surgery (5/5) | Multiple (unspecified) | 3 months | 3 months of physiotherapy | No | Low |
| 24. Mikami T, 2012 [41] | 3 | 30, 31, 59 | 3 F | 3 L | Pain (3/3), reduced MIO (3/3), click (1/3), swelling (1/3) | N.S. | N.S. | 6 months, 1.5 years, 2.5 years | OPT, CT, MRI | Open Surgery (3/3) | Approximately 100 (2/3), N.S. (1/3) | 28 months, 33 months, 38 months | N.S. | No | High |
| 25. Wang P, 2012 [42] | 22 | mean 45.3 years, range (17–64) | 15 F, 7 M | L (10/22) R (12/22) | - | - | - | - | MRI | - | - | - | - | - | High |
| 26. Wake M, 2012 [43] | 10 | N.S. | 6 F, 4 M | N.S. | Pain (10/10), reduced MIO (10/10), grade 3 joint effusion (10/10) | No | No | N.S. | MRI | Arthroscopy and visually guided irrigation (7/10), Open Surgery (3/10) | N.S. | N.S. | N.S. | N.S. | High |
| 27. Kahraman AS, 2012 [44] | 1 | 49 | 1 M | 1 R | Reduced MIO | No | N.S. | 2.5 years | CT, MRI | N.S. | Multiple (unspecified) | No | N.S. | N.S. | High |
| 28. Matsumura Y, 2012 [45] | 1 | 46 | 1 M | 1 R | Pain, reduced MIO | No | N.S. | N.S. | CT, MRI | N.S. | 1 (10 × 10 × 8 mm) | No | N.S. | No | High |
| 29. van Dijk RR, 2012 [46] | 1 | 70 | 1 F | - | - | - | - | - | CT, MRI | - | - | - | - | - | High |
| 30. Yoshida H, 2013 [47] | 4 | - | 4 F | - | - | - | - | - | - | - | - | - | - | - | High |
| 31. Coleman H, 2013 [48] | 1 | 63 | 1 F | 1 L | Swelling | No | N.S. | N.S. | OPT, CT, MRI | Open Surgery with soft tissue free flap | 1 (50 × 55 × 40 mm) | N.S. | N.S. | N.S. | High |
| 32. Zhu Y, 2013 [49] | 1 | 21 | 1 F | 1 L | Pain, reduced MIO | No | N.S. | N.S. | CT, MRI | Arthroscopy | 112 (0.5 to 6.5 mm) | 3 months | N.S. | No | High |
| 33. Reed LS, 2013 [50] | 1 | 31 | 1 F | 1 R | Pain, swelling | No | N.S. | 3–4 months | MRI | Open Surgery | Multiple (unspecified) | 3 months | N.S. | No | Unclear |
| 34. Pau M, 2014 [51] | 1 | 70 | 1 M | 1 R | Pain, swelling upon mouth opening, headache while chewing | N.S. | N.S. | 5 years | OPT, CT, MRI | Open Surgery with diskectomy and capsulotomy | 1 | 6 months | N.S. | No | High |
| 35. Peyrot H, 2014 [52] | 1 | 42 | 1 F | 1 L | Pain, swelling, crepitus | No | No | N.S. | OPT, CT, MRI | Open Surgery | N.S. | N.S. | N.S. | No | High |
| 36. Sink J, 2014 [53] | 1 | 82 | 1 M | 1 L | Alzheimer disease and temporal lobe epilepsy | N.S. | N.S. | N.S. | MRI | Open Surgery | Multiple (unspecified) | N.S. | N.S. | N.S. | High |
| 37. Li Y, 2014 [54] | 3 | - | - | - | - | - | - | - | - | - | - | - | - | - | High |
| 38. Valentini V, 2014 [55] | 1 | 60 | 1 F | 1 R | Pain, swelling | No | No | Many years (unspecified) | CT, MRI | Open Surgery with minimal remodeling of glenoid fossa | Multiple (unspecified) | N.S. | N.S. | No | High |
| 39. Cascone P, 2014 [56] | 1 | 30 | 1 M | 1 L | Pain, reduced MIO | No | No | 6 months | MRI | Arthroscopy | 11 (20–30 mm) | N.S. | N.S. | N.S. | High |
| 40. Droguett C, 2014 [57] | 1 | 49 | 1 F | 1 R | Pain (VAS = 8), reduced MIO (15 mm), swelling | N.S. | No | N.S. | MRI | Open Surgery with meniscopexy | Multiple (unspecified) | 12 months | N.S. | No | High |
| 41. Chen MJ, 2015 [58] | 144 | Mean 48.6, range 17–73 | M/F ratio: 1/2.35 | R/L 1/1.18 | - | - | - | - | MRI | Open Surgery (105/144) Arthroscopy (39/144) | Multiple (unspecified) | - | - | - | High |
| 42. Pinto AA Jr, 2015 [59] | 1 | 54 | 1 F | 1 L | Pain, reduced MIO (20 mm), swelling | No | N.S. | 1 year | CT, MRI | Open Surgery | 11 (10 mm) | 36 months | N.S. | No | Unclear |
| 43. Ochoa Escudero M, 2015 [60] | 1 | 63 | 1 M | - | - | - | - | - | CT, MRI | - | - | - | - | - | High |
| 44. Wang Y, 2015 [61] | 133 | - | - | - | - | - | - | - | - | - | - | - | - | - | High |
| 45. Ivask O, 2015 [62] | 1 | 45 | 1 F | 1 L | Reduced MIO (38 mm), hearing disturbances, preauricular tenderness, click | No | No, but history of arthrosis | N.S. | OPT, CT | Open Surgery | Multiple (unspecified), 3–10 mm diameter | 6 months | Post-operative anti-inflammatory and analgesic drugs and physiotherapy | No | Unclear |
| 46. Ionna F, 2016 [63] | 1 | 62 | 1 F | 1 R | Pain, reduced MIO (20 mm), swelling | N.S. | N.S. | 5 months | CT, MRI | Transoral surgical approach | 1 | 3 months | N.S. | No | Unclear |
| 47. Pastore GP, 2016 [64] | 1 | 30 | 1 F | 1 R | Pain, reduced MIO (27 mm), bilateral click | No | No | 6 months | MRI, CT | Arthroscopy | 1 (0.6 × 0.4 cm) | 12 months | Infiltration with hyaluronic acid 30 and 60 days after surgery | No | Low |
| 48. Wake S, 2016 [65] | 17 | 8.5 ± 14.14 years (range 28–71) | 3 M, 14 F | - | - | - | - | - | - | - | - | - | - | - | High |
| 49. Yoshitake H, 2016 [66] | 1 | 72 | 1 M | 1 R | Pain, reduced MIO (30 mm), swelling, tenderness | No | No | Several years (unspecified) | CT, MRI | Open Surgery | 1 | 84 months | N.S. | No | Low |
| 50. Sozzi D, 2016 [67] | 1 | 68 | 1 F | 1 R | Pain, reduced MIO (20 mm), swelling | No | No | 4 months | CT, MRI | Open Surgery with partial synovectomy | Multiple (unspecified) | 36 months | Physiotherapy for 45 days | No | Low |
| 51. Paparo F, 2016 [68] | 1 | 36 | 1 F | 1 R | Pain, reduced MIO (12 mm), swelling | N.S. | N.S. | N.S. | OPT, CT, MRI | Arthroscopy | 22 | 6 months | N.S. | No | High |
| 52. Liu X, 2016 [69] | 10 | 45.4 ± 9.4 | 8 F, 2 M | 2 R 8 L | Pain (7/10), reduced MIO (7/10), swelling (7/10), crepitus (5/10), | Yes (2/10) | No | N.S. | CBCT (9/10), CT (1/10) | Open Surgery (10/10) | Multiple (unspecified) | 2 to 52 months | N.S. | No | High |
| 53. Selvi F, 2016 [70] | 1 | 45 | 1 F | 1 R | Pain, reduced MIO swelling, tenderness | No | Yes | 18 months | OPT, MRI | Arthroscopy | Multiple (unspecified) | 6 months | N.S. | No | Low |
| 54. Leite PCC, 2017 [71] | 1 | 56 | 1 F | 1 R | Pain, click, crepitus, clenching | No | No | 2 years | CT, MRI | Open Surgery with diskectomy and condyloplasty. Disc replaced with muscle flap from temporalis muscle and fascia | N.S. | 12 months | N.S. | No | Low |
| 55. Pappot TW, 2017 [72] | 1 | 54 | 1 F | 1 L | Pain, reduced MIO, swelling, tenderness | No | N.S. | 10 years | OPT, CT, MRI | Open Surgery | Multiple (unspecified), 1–5 mm diameter | N.S. | N.S. | N.S. | High |
| 56. Bai G, 2017 [73] | 36 | Mean 48.11 range (29–65) | 25 F, 11 M | 14 R 22 L | Pain (36/36), reduced MIO (36/36), joint sound (5/36), click (4/36) | N.S. | N.S. | 27.19 months (range 1–20) | MRI (36/36) | Open surgery assisted with arthroscopy, meniscopexy (36/36) | Multiple (unspecified) | 60 months (33/36), no (3/36) | N.S. | No | High |
| 57. Wilms CT, 2017 [74] | 16 | Mean age 54 years (range 36–71) | 11 F, 5 M | - | - | - | - | - | - | - | - | - | - | - | High |
| 58. McCaffery C, 2017 [75] | 1 | 34 | 1 M | 1 R | Swelling | Yes | No | 5 years | OPT, CT, MRI | Open Surgery | 1 | N.S. | N.S. | N.A. | High |
| 59. Khanna JN, 2017 [76] | 2 | 43, 35 | 2 M | 1 R 1 L | Pain (2/2), reduced MIO (2/2), swelling (2/2) | N.S. | N.S. | 2 years (1/2), 1 year (1/2) | CT, MRI (2/2) | Open Surgery (2/2) | 1 (2/2) | 108 months (1/2), 36 months (1/2) | N.S. | No | Unclear |
| 60. Han ZX, 2017 [77] | 2 | 51, 48 | 1 F, 1 M | - | Pain (2/2), reduced MIO (2/2) | - | - | - | CT, MRI (2/2) | Open Surgery (synovectomy, condylotomy, TMJ reconstruction) (1/2), synovectomy, resection of the articular eminence, partial osteotomy of the fossa and reconstruction of the skull base with a pedicled deep temporal fascial fat flap (1/2) | Multiple (unspecified) | N.S. | N.S. | No | High |
| 61. Kim DH, 2017 [78] | 2 | 26, 31 | 2 F | 1 R 1 L | Pain (2/2) | N.S. | N.S. | N.S. | CT, MRI | Open Surgery (2/2) | Multiple (unspecified) | 30 months (1/2), 6 months (1/2) | N.S. | No | High |
| 62. Holtmann H, 2018 [79] | 1 | 63 | 1 F | 1 R | Pain, swelling | No | N.S. | 2 years | OPT, CT, MRI | Open Surgery | 25 | N.S. | N.S. | N.S. | High |
| 63. Kim HS, 2018 [80] | 1 | 50 | 1 M | 1 L | Pain, swelling | N.S. | N.S. | N.S. | OPT, CT, MRI | Open Surgery | 120 approximately | 3 months | N.S. | No | High |
| 64. Singh K, 2018 [81] | 1 | 59 | 1 F | 1 R | Pain, swelling, click | N.S. | N.S. | 15 days | OPT, CT, MRI | Refused surgical intervention | NA | NA | NA | NA | High |
| 65. Romano A, 2018 [82] | 1 | 45 | 1 F | 1 R | Swelling, click, crepitus, headache while eating | No | N.S. | 5 years | OPT, CT, MRI | Open Surgery with capsulotomy | Multiple (unspecified) | 28 months | N.S. | No | Low |
| 66. Vellone V, 2018 [83] | 1 | 64 | 1 F | 1 R | Pain, swelling, reduced MIO (15 mm) | No | No | N.S. | CT, MRI, Echography | Open Surgery | 1 (3.5 × 1 × 0.8 mm) | 24 months | N.S. | No | Low |
| 67. Mikami T, 2018 [84] | 1 | 40 | 1 F | 1 R | Pain, reduced MIO (34 mm), tenderness | No | No | 2 years | CT, MRI | Open Surgery | 1, 25 mm in diameter | 36 months | N.S. | No | Low |
| 68. Brabyn PJ, 2018 [85] | 6 | 38, 37, 33, 44, 43, 57 | 5 F, 1 M | 6 L | Pain (6/6), reduced MIO (5/6) swelling (2/6) | No (6/6) | No (6/6) | N.S. | CT, MRI (6/6) | Arthroscopy (6/6) | Multiple (unspecified) (0.5–3 mm) | 12 months | Physiotherapy | No | Low |
| 69. Benslama L, 2019 [86] | 12 | Mean age 50.5, range 43–86 | 8 F, 4 M | 9 L 3 R | Pain (10/12), reduced MIO (6/12), swelling (4/12), crepitus (2/12), tinnitus (2/12) | No | N.S. | Average 11 months, range 1–24 | OPT, CT, MRI (12/12) | Open Surgery (11/12) Arthroscopy (1/12) | 2 to 30 | Average 78 months (range 54–118) | N.S. | No (10/12), Yes (1/12), lost at follow-up (1/12) | Low |
| 70. Lee LM, 2019 [87] | 16 | Mean age 32.68 (range 19 to 49) | 2F, 14 M | 7 R 9 L | Pain (9/16), reduced MIO (16/16), crepitus (8/16) | No | N.S. | N.S. | MRI (15/16), CT (2/16) | Arthroscopy (16/16) | Multiple (40 to 100) | 36 months | N.S. | No | Low |
| 71. Maffia F, 2019 [88] | 6 | Mean age 48.6, range 30 to 61 | 4 F, 2 M | 4 L 2 R | Pain (6/6), reduced MIO (5/6), swelling (5/6), crepitus (3/6) | N.S. | N.S. | Average 4 years (range 3–5) | CT, MRI | Open Surgery (6/6) | Multiple (unspecified) | 12 months | N.S. | No | High |
| 72. Tang B, 2019 [89] | 3 | 60, 38, 30 | 3 F | 2 R 1 L | Tenderness (1/3), swelling (2/3) | No (3/3) | Yes (1/3) No (2/3) | 3 years, 6 months, 2 months | CT (3/3) | Open Surgery with synovectomy (3/3) | Multiple (unspecified) | N.S. | N.S. | N.S. | High |
| 73. Liu X, 2019 [90] | 156 | - | - | - | - | - | - | - | MRI (156/156) | - | - | - | - | - | High |
| 74. Matsuda S, 2019 [91] | 1 | 73 | 1 M | 1 L | Pain, swelling | No | N.S. | 25 years | OPT, CT, MRI | Open Surgery | Multiple (unspecified) | 30 months | N.S. | No | Unclear |
| 75. Mathew P, 2019 [92] | 1 | 36 | 1 M | 1 L | Pain, reduced MIO (33 mm), swelling, tenderness | N.S. | N.S. | 2 weeks | OPT, CT, MRI | Open Surgery | 1 | N.S. | N.S. | N.S. | High |
| 76. Barraclough O, 2020 [93] | 1 | 32 | 1 F | 1 L | Pain | N.S. | N.S. | N.S. | OPT, CT | Open Surgery | Multiple (unspecified) | N.S. | Physiotherapy | N.S. | High |
| 77. Haapalainen L, 2020 [94] | 1 | 49 | 1 M | 1 R | Pain, reduced MIO, swelling | Yes (20 years before) | N.S. | N.S. | OPT, MRI | Open Surgery | Multiple (unspecified) | N.S. | N.S. | N.S. | High |
| 78. Nath P, 2020 [95] | 1 | 38 | 1 M | 1 R | Pain, reduced MIO (38 mm), swelling, tenderness | No | N.S. | 8 years | OPT, CBCT, CT | Open Surgery with osteotomy of condyle neck and meniscopexy | Multiple (unspecified) | 36 months | N.S. | No | High |
| 79. Gross AJ, 2020 [96] | 1 | 68 | 1 F | 1 L | Reduced MIO, swelling (1 cm) | No | No but osteoarthritis | Incidental diagnosis | CT | Open Surgery with discectomy | 1 | 2 months | N.S. | No | Low |
| 80. Morales RJ, 2020 [97] | 1 | 56 | 1 F | 1 R | Pain | No | No | 6 years | OPT, CT | Open surgery with synovectomy and meniscopexy | 1 (10 × 4 mm) | 36 months | Physiotherapy | No | Low |
| 81. Barretto MDA, 2020 [98] | 1 | 60 | 1 F | Bilateral | Pain, reduced MIO (25 mm) | No | No but osteoarthritis of knee | 8 years | OPT, CT | Open Surgery | Multiple (unspecified) | 13 months | N.S. | No | Low |
| 82. Yoshida H, 2020 [99] | 2 | - | 2 F | - | - | - | - | - | - | - | - | - | - | - | High |
| 83. Jang BG, 2021 [100] | 34 | Mean age 48.8 (range 23–73) | 28 F 6M | 16 L 18 R | Pain (31/34), swelling (5/34), trismus (11/34) | No | No | N.S. | CT, MRI | Open Surgery (21/34), observation (13/34) | Multiple (unspecified) | N.A. | N.S. | N.A. | High |
| 84. Zhao W, 2021 [101] | 1 | 53 | 1 F | 1 L | Pain, reduced MIO, swelling, click | No | No | 5 months | CT | Open Surgery with partial synovectomy | Up to 400 | 108 months | N.S. | No | Low |
| 85. Yoshitake H, 2021 [102] | 1 | 67 | 1 F | 1 R | Pain, reduced MIO (25 mm), tenderness | No | No | N.S. | MRI | Open Surgery | 1 | N.S. | N.S. | N.S. | High |
| 86. Nishiyama M, 2021 [103] | 11 | Mean age 52.1 years | 7 F, 4 M | 6 L 5 R | Pain (8/11), reduced MIO (6/11), swelling (4/11), crepitus (7/11) | N.S. | N.S. | Unknown (3/12), 12 months (2/12), 24 months (1/12), 36 months (1/12), 30 months (1/12), 6 months (1/12), 3 months (1/12), 1 month (1/12) | OPT, CT (12/12) | - | - | - | - | - | High |
| 87. Destruhaut F, 2021 [104] | 1 | 21 | 1 F | 1 L | Pain, reduced MIO (31mm) | Yes (whiplash) | Yes | 2 years | OPT, CBCT | Open Surgery with mandibular condyloplasty and a temporal condylotomy | 30 (1–7 mm) | N.S. | Physiotherapy and occlusal splint | N.S. | Low |
| 88. Durna D, 2021 [105] | 1 | 53 | 1 F | 1 R | Pain, reduced MIO | No | N.S. | N.S. | OPT, CT, MRI | - | - | - | - | - | High |
| 89. Cameron A, 2021 [106] | 1 | 52 | 1 F | 1 L | - | - | - | - | - | - | - | - | - | - | High |
| 90. Matsusue Y, 2021 [107] | 1 | 56 | 1 F | 1 L | Pain, reduced MIO (30 mm), trismus | N.S. | N.S. | 2 years | CT, MRI | Open Surgery, condyloplasty | Multiple 386, 2–5 mm in diameter | 60 months | N.S. | No | Low |
| 91. Fukutani T, 2022 [108] | 2 | 38, 39 | 2 F | 1 L 1 R | Pain (2/2), reduced MIO (2/2), noise (2/2), crepitus, mandibular shift to right (1/2), mandibular shift to left (1/2) | No | No | 11 years (1/2), 5 years (1/2) | CT, MRI (2/2) | Open Surgery (2/2) | 151 (1/2), multiple unspecified (1/2) | 96 months (1/2), 72 months (1/2) | Physiotherapy + splint | No | Low |
| 92. Satyanarayan P, 2022 [109] | 5 | Mean age 36.4, range 30–42 | 3 M, 2 F | N.S. | Pain (5/5), reduced MIO (5/5) | Yes (3/5) | No | N.S. | MRI | Arthroscopy (5/5) | Multiple (unspecified) | 12 months | N.S. | No | Low |
| 93. Jia M, 2022 [110] | 14 | 57, 63, 50, 70, 44, 62, 32, 54, 70, 58, 40, 52, 43, 43. Mean age 52.7 (range 32–70) | 13 F, 1 M | 7 L 7 R | Pain (11/14), reduced MIO (4/14), swelling (3/14), click (1/14), noise (6/14) | N.S. | N.S. | 10 years (2/14), 6 years (2/14), 4 years (1/14), 3 years (1/14), 2 years (3/14), 1 year (1/14), 6 months (1/14), 2 months (1/14), 2 weeks (1/14), incidental diagnosis (1/14) | MRI | Open Surgery (14/14) | Multiple (unspecified) | 6 to 48 months | N.S. | No | Low |
| 94. Nashi M, 2022 [111] | 1 | 58 | 1 M | 1 R | Pain, reduced MIO (20 mm), trismus | No | No | 3 years | OPT, CT, MRI | Open Surgery | Multiple (unspecified) | N.S. | N.S. | N.A. | High |
| 95. Marahashi M, 2022 [112] | 1 | 61 | 1 M | 1 R | Pain, reduced MIO | No | Yes | 6 months | OPT, CT, MRI | Open Surgery | Multiple (unspecified) | 18 months | N.S. | No | Low |
| 96. Song Z, 2022 [113] | 7 | 45.14 ± 11.88 | 5 F, 2 M | 1 L 6 R | Pain (7/7), reduced MIO (4/7), swelling (1/7), click (1/7), crepitus (3/7) | No | N.S. | 5 years (1/7), 3 years (1/7), 2 years (2/7), 1 years (1/7), 6 months (1/7), 1 month (1/7) | CBCT, MRI | Open Surgery (6/7), arthroscopy assisted open surgery (1/7) | N.S. | N.S. | N.S. | N.S. | High |
| 97. Burris B, 2023 [114] | 1 | 34 | 1 M | 1 R | Pain, mandible deviation | No | No | 4 years | OPT, CT, MRI | Arthroscopy | N.S. | No | N.S. | N.A. | High |
| 98. Han W, 2023 [115] | 100 | Median 47 (range 21–77) | 75 F, 25 M | 40 L 59 R 1 bilateral | Pain (50/100), reduced MIO (19/100), swelling (50/100), click (8/100), mandibular deviation (2/100), tinnitus and hearing loss (3/100) | Yes (3/100) | N.S. | 15 days to 10 years (median 23.5 months) | CBCT, MRI | Arthroscopy (7/100), Open surgery (93/100) | N.S. | 8 months to 228 months | N.S. | Yes (9/100) | High |
| 99. Kaneko I, 2023 [116] | 1 | 56 | 1 M | 1 L | Pain, click | No | No |  | OPT, CT, MRI | Open Surgery with condylectomy | Multiple (unspecified) | 36 months | No | No | Low |
| 100. Jenser AC, 2023 [117] | 2 | 58, 63 | 1 F, 1 M | 2 R | Pain (1/2), reduced MIO (1/2), osteoarthritis (1/2) | No | N.S. | 25 years (1/2), incidental diagnosis (1/2) | OPT, CT (1/2), OPT, CT, MRI (1/2) | Open Surgery (2/2) | 27 (1/2), multiple (1/2) | 72 months (1/2), N.S. (1/2) | No | No (1/2), N.A. (1/2) | High |
| 101. Machado GG, 2023 [118] | 3 | 55, 57, 48 | 3 F | 2 L 1 R | Pain (2/3), reduced MIO (2/3), swelling (3/3), no history of trauma (3/3) | N.S. | N.S. | 5 years, 3 years, 2 years | CT, MRI | Open Surgery (3/3) | N.S. | 60 months, 48 months, 36 months | N.S. | No | High |
| 102. Cini MA, 2023 [119] | 1 | 37 | 1 M | 1 L | Pain | N.S. | N.S. | 1 year | MRI | Arthroscopy | 34 | 6 months | N.S. | No | High |
| 103. Morishima H, 2023 [120] | 1 | 49 | 1 F | 1 L | Pain, reduced MIO (32 mm), crepitus | N.S. | N.S. | 2 years | OPT, CT, MRI | Arthroscopy | Multiple (unspecified) | N.S. | N.S. | N.A. | High |
| 104. Jang BG, 2023 [121] | 35 | - | - | - | - | - | - | - | - | - | - | - | - | - | High |
| 105. Sembronio S, 2023 [122] | 16 | Mean 50.9, range 24–74 | 13 F, 3 M | 10 L 6 R | Pain (16/16) reduced MIO (16/16) (range 20–38 mm, median 37.9) | N.S. | N.S. | N.S. | MRI (16/16), CT (5/16) | Arthroscopy (14/16), Open Surgery (2/16) | Multiple (unspecified) | 6 months | N.S. | Yes (2/16), No (14/16) | Low |
| 106. Zhang Y, 2024 [123] | 38 | Mean age 53.2 ± 12.6, range 26–72 | 27 F, 13 M | 18 L 20 R | Pain (31/38), reduced MIO (29/36) (30–38 mm), swelling (24/38) | N.S. | N.S. | N.S. | CT, CBCT | Open Surgery (38/38) | - | - | - | - | High |
| 107. Vladimír M, 2024 [124] | 37 | Average age 54.77, range 20 to 86 | 26 F, 11 M | 23 R 13 L 1 bilateral | Pain (32/37), reduced MIO <30 mm (8/37), swelling (6/37), crepitus (3/37) | N.S. | N.S. | N.S. | OPT, CT, MRI | Arthroscopy (21/37), open surgery (14/37) | N.S. | N.S. | N.S. | Yes (4/37), No (33/37) | High |
| 108. Spallaccia F 2024 [125] | 1 | 44 | 1 F | 1 L | Pain (VAS 6), reduced MIO (27 mm), swelling | N.S. | No | N.S. | PETRA MRI | Arthroscopy | N.S. | 12 months | N.S. | No | High |

Legends: M, male; F, female; R, right; L, left; MIO, mouth interincisal opening; OPT, orthopantomography; MRI, magnetic resonance imaging; CT, computer tomography; CBCT, cone beam computerized tomography; NSAID, Non-steroidal anti-inflammatory drugs; N.S., Not specified; N.A., Not available; mm, millimeters; VAS, Visual Analog Scale.
